# Revision of the ant-eating spider genus *Mallinus* Simon, 1893 (Araneae, Zodariidae)

**DOI:** 10.3897/zookeys.822.29835

**Published:** 2019-02-06

**Authors:** Charles R. Haddad, Arnaud Henrard, Rudy Jocqué

**Affiliations:** 1 Department of Zoology & Entomology, University of the Free State, P.O. Box 339, Bloemfontein 9300, South Africa University of the Free State Bloemfontein South Africa; 2 Department of Zoology, Royal Museum for Central Africa, Leuvensesteenweg 13, B-3080 Tervuren, Belgium Royal Museum for Central Africa Tervuren Belgium; 3 Earth and life Institute, Biodiversity Research Center, Université catholique de Louvain, B-1348, Louvain-la-Neuve, Belgium Université catholique de Louvain Louvain-la-Neuve Belgium

**Keywords:** Arid, endemic, myrmecophagous, Nama Karoo, South Africa, Zodariinae

## Abstract

The zodariine spider genus *Mallinus* Simon, 1893 is redescribed and diagnosed. The type species, *M.nitidiventris* Simon, 1893 from South Africa, was originally described from subadult specimens. Adults of both sexes of *M.nitidiventris* are described for the first time, based on recently collected material, and the genus is rediagnosed, redescribed, and its relationships discussed. A single aberrant male specimen from Namibia is here described as a morphospecies, as it is presumed to only be superficially related. A second species, *M.defectus* Strand, 1906 from Tunisia, is considered a ‘*species inquirenda*’, as the type specimens could not be traced, but this species is in any case unlikely to be congeneric. The genus is one of 10 cases of a monotypic genus in the Zodariidae. Notes are provided on the biology of *M.nitidiventris*.

## Introduction

The Zodariidae is a medium-sized family of spiders, with 1140 species in 85 genera globally (World Spider Catalog 2018). The subfamily Zodariinae is of particular interest from a biological perspective, as most of the species are exclusively myrmecophagous (Cushing 2012, Pekár et al. 2012, Pekár and Toft 2015, Komatsu 2016), which reflects its derived phylogenetic position in the family (Jocqué 1991).

The genus *Mallinus* Simon, 1893 remains one of the most poorly understood genera of Zodariinae. Initially described from a single South African locality, the type species (*M.nitidiventris* Simon, 1893) has never been properly redescribed, as the type series consists of a subadult male and subadult female (Jocqué 1991), not adult specimens as indicated by Simon (1893). A second species, *M.defectus* Strand, 1906, was described from Tunisia (Strand 1906) in the Palaearctic Region, but it was doubtfully placed in *Mallinus* originally, and as explained in the discussion it is unlikely to belong to this genus. Simon (1893) placed *Mallinus* in his “Zodarieae” together with *Zodarion* Walckenaer, 1826 and *Diores* Simon, 1893, although the concept of this subfamily has expanded significantly since then through a series of revisions led by the third author (Jocqué 1991; World Spider Catalog 2018).

The recent collection of adult specimens in South Africa fitting Jocqué’s (1991) illustrations of *M.nitidiventris* served as the impetus to redescribe the type species and assess whether other congeneric species may occur in Africa. Examination of museum collections yielded several additional records of *M.nitidiventris*, indicating a surprisingly broad distribution of the species in the arid Nama Karoo Biome of South Africa, but also extending into the Succulent Karoo and the arid parts of the Savanna Biome. A revised diagnosis and description, aided by scanning electron micrographs, are provided to recognize *Mallinus* from other Zodariinae spiders.

A single male from northern Namibia is here described as “*Mallinus*” sp. Although the shape and the texture of the cephalothorax and the abdomen are similar to that of *M.nitidiventris*, we suppose that it belongs to a different genus, mainly due to the absence of a conductor on the male palp and the extremely unusual eye pattern: the AME are much larger than the remainder and the ALE are very wide apart, situated in the far lateral corners of the clypeus. However, we have refrained from describing a new genus on the base of a single male, and await further material to place it.

## Materials and methods

The specimens examined in the current study were preserved in 70% ethanol and examined using a Nikon SMZ800 stereomicroscope for measurements and descriptions. The female genitalia and male palps were drawn with a WILD M10 stereomicroscope (Leica). The female genitalia were then dissected and digested using half a tablet of Total Care Enzima product (protein removal system originally for cleaning contact lenses and containing subtilisin A-0.4 mg per tablet; Abbott Medical Optics, Santa Ana, CA) in a few millilitres of distilled water overnight, and then immersed in 75% ethanol. These female genitalia and male palps were photographed with a Leica MZ16 using the Leica Application Suite (LAS) automontage software (ver. 3.8; Leica, https://leicacamera.com).

All measurements are given in millimetres (mm). Measurements of somatic morphological structures were taken from one specimen of each sex, as indicated, while total length measurements were taken for all available specimens to determine size variation. Leg lengths are presented as the sequence from femur to tarsus, and total. Digital photographs of the dorsal and lateral habitus of both sexes of *M.nitidiventris* were taken with a Nikon D5-L3 camera system attached to a Nikon SMZ800 stereomicroscope. To increase depth of field, a series of images was taken and stacked using the CombineZM imaging software (http://www.hadleyweb.pwp.blueyonder.co.uk).

Material for scanning electron microscopy (SEM) was freshly collected from the farm Bankfontein in the western Free State, South Africa (see Material examined) and immediately preserved in 100% ethanol (see below). Prior to SEM, material was transferred to fresh 100% ethanol overnight, critical point dried in an argon chamber, glued to aluminium stubs using double-sided tape, and sputter coated with gold. Somatic and genitalic structures were examined in a JEOL JSM-7800F FE-SEM at 3 kV and digital photographs were taken.

The following abbreviations are used in the descriptions: **AER** – anterior eye row; **AH** – abdomen height; **AL** – abdomen length; **ALE** – anterior lateral eye; **ALS** – anterior lateral spinneret; **AME** – anterior median eye; **AW** – abdomen width; **CL** – carapace length; **CW** – carapace width; **F** – femur; **FL** – fovea length; **imm.** – immature; **MA** – median apophysis; **MOQ** – median ocular quadrangle; **MOQAW** – median ocular quadrangle anterior width; **MOQL** – median ocular quadrangle length; **MOQPW** – median ocular quadrangle posterior width; **PER** – posterior eye row; **PERW** – posterior eye row width; **PLE** – posterior lateral eye; **PLS** – posterior lateral spinneret; **PME** – posterior median eye; **PMS** – posterior median spinneret; **RTA** – retrolateral tibial apophysis; **SL** – sternum length; **ST** – spermatheca; **SW** – sternum width; **T** – tibia; **TL** – total length; **v** – ventral.

The examined material was obtained from the Muséum National d’Histoire Naturelle, Paris, Frances (**MNHN**, Christine Rollard), Royal Museum for Central Africa, Tervuren, Belgium (**MRAC**, Rudy Jocqué), State Museum of Namibia, Windhoek (**SMN**, Eryn Griffin) and the National Collection of Arachnida, ARC – Plant Protection Research, Pretoria, South Africa (**NCA**, Petro Marais).

## Taxonomy

### Family Zodariidae

#### Genus Mallinus

Taxon classificationAnimaliaAraneaeZodariidae

Simon, 1893

Mallinus Simon, 1893: 436; Jocqué 1991: 136.

##### Type species.

*Mallinusnitidiventris* Simon, 1893, by monotypy.

##### Diagnosis.

*Mallinus* can be distinguished from other zodariine spiders by the relatively smaller size of the anterior median eyes, which are only slightly larger than the lateral and posterior eyes, while generally much larger than the other eyes in other zodariines. *Mallinus* shares with *Palfuria* Simon, 1910 the scale-like extensions on the endites and the considerably raised cephalic region, but lacks the carapace modifications at the posterior end of the cephalic region typical for most *Palfuria* (Szüts and Jocqué 2001); rather, the carapace slopes steeply at the posterior end of the cephalic region, with only a shallow transverse depression. *Mallinus* also have a very globose abdomen, which is usually higher than long in both sexes, a rare condition amongst other zodariines.

##### Description.

**Figures 1–4.**
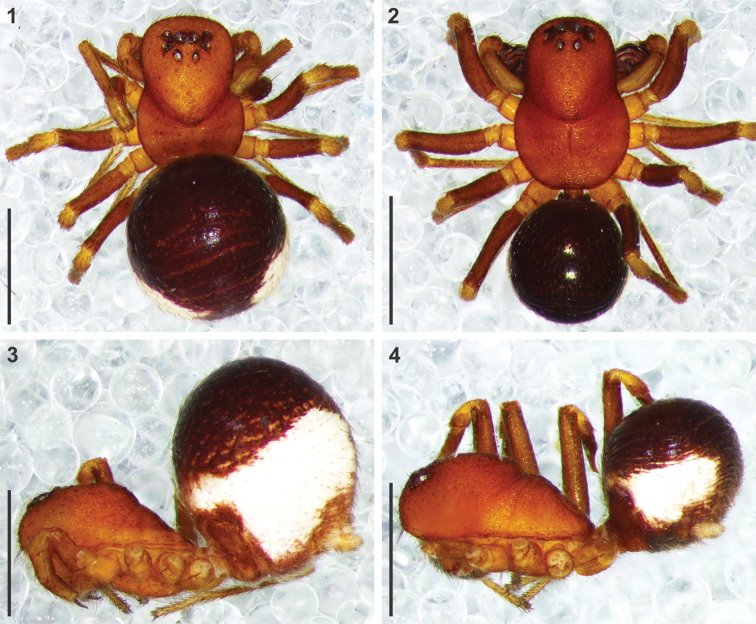
Somatic morphology of *Mallinusnitidiventris* female (**1, 3**) and male (**2, 4**) from Bankfontein, Free State (NCA 2015/1818) **1, 2** habitus, dorsal view **3, 4** same, lateral view. Scale bars: 1.0 mm.

**Figures 5–19.**
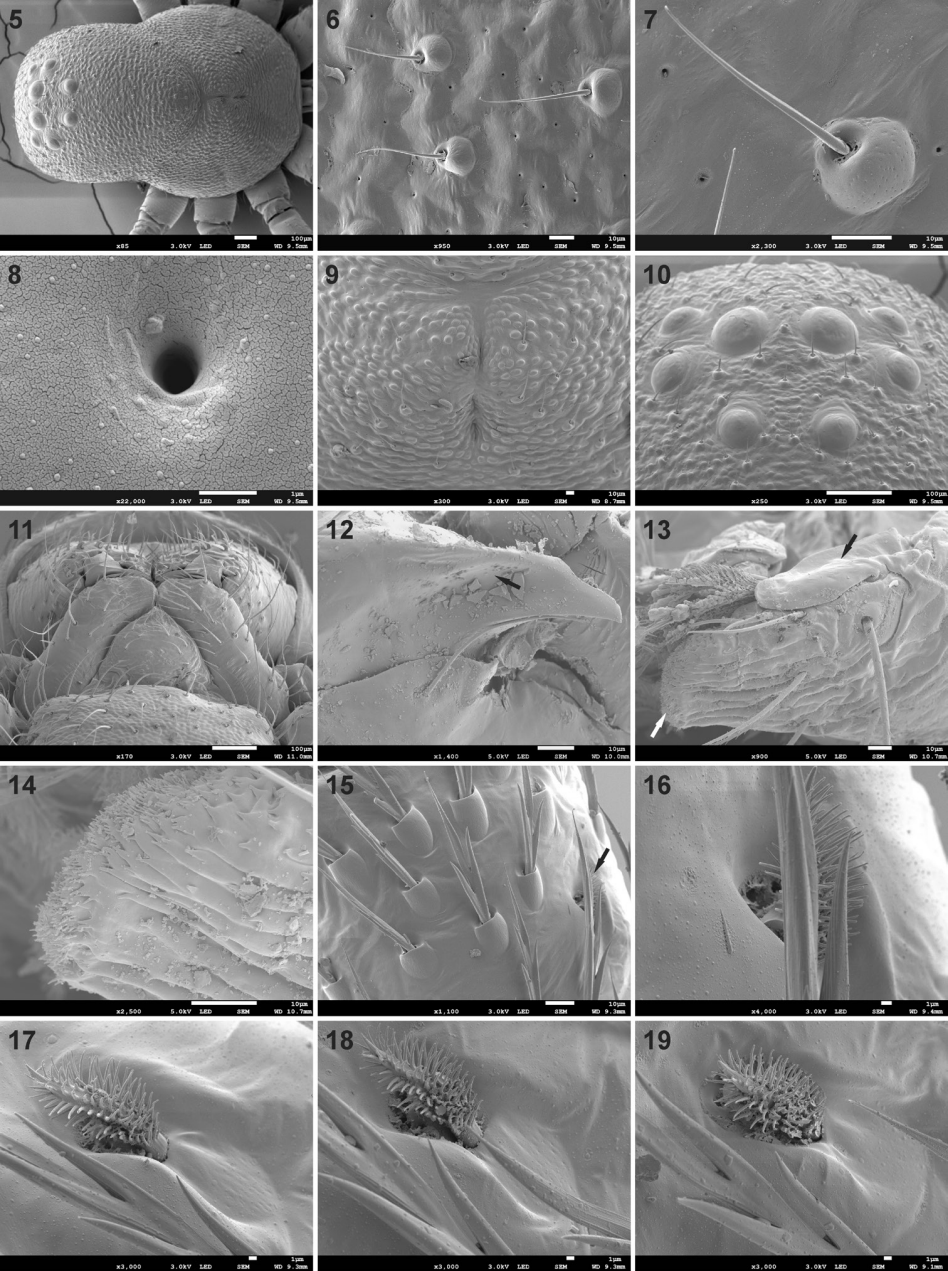
Scanning electron microscope photographs of *Mallinusnitidiventris* male (**5–10, 12–19**) and female (**11**) **5** carapace, dorsal view **6** detail of carapace integument **7** same, detail of carapace setae **8** same, detail of carapace pore **9** fovea **10** eye region, dorsal view **11** endites, labium and anterior end of sternum **12** distal end of chelicera, arrow indicating posterior groove of fang **13** distal end of endite, black arrow indicating distal endite scale, white arrow indicating prolateral field of flattened denticles **14** prolateral margin of endite, showing detail of flattened denticles **15** femur I, dorsal view, incised setae and femoral organ (black arrow) **16–19** femoral organ on legs I–IV, respectively.

**Figures 20–34.**
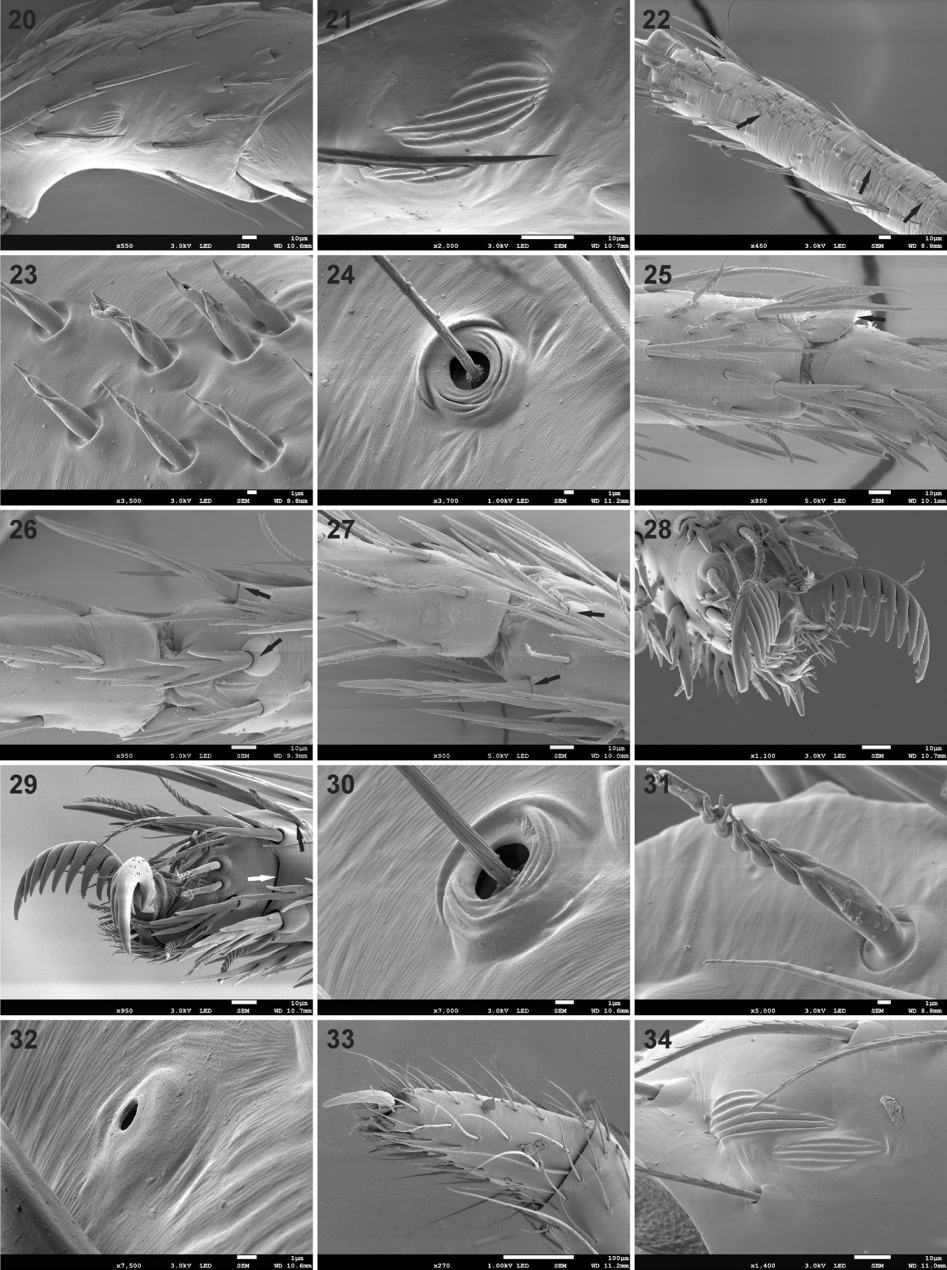
Scanning electron microscope photographs of *Mallinusnitidiventris* female (**20, 21, 28, 29, 33, 34**) and male (**22–27, 30–32**) **20** patella IV, retrolateral view **21** same, detail of lyriform organ **22** metatarsus I, incised setae and dorsal fields of short chemosensory setae (black arrows) **23** same, detail of short chemosensory setae **24** metatarsus I, trichobothrium **25** metatarsus III, arrow indicating metatarsal stopper **26** metatarsus III, arrows indicating thickened ventral terminal setae **27** same, metatarsus IV **28** tarsus I, paired claws in distal view **29** same, prolateral distal view, black arrow indicating chemosensory seta, white arrow indicating subdistal suture of tarsus **30** same, trichobothrium **31** same, chemosensory seta **32** same, tarsal organ **33** palpal tarsus and claw **34** palpal patella, lyriform organ.

**Figures 35–46.**
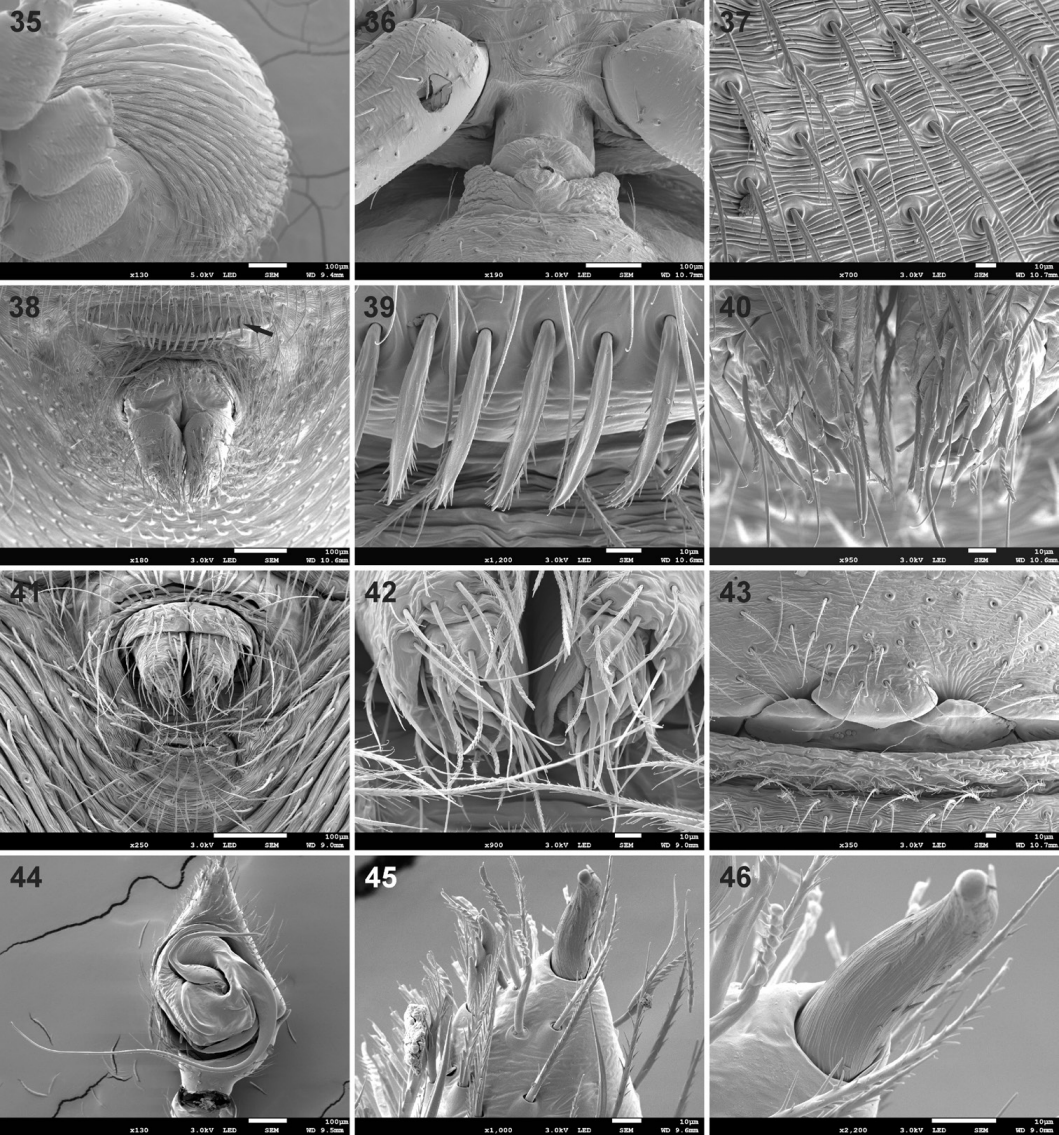
Scanning electron microscope photographs of *Mallinusnitidiventris* male (**35, 41, 42, 44–46**) and female (**36–40, 43**) **35** abdomen, ventrolateral view **36** petiole, ventral view **37** ventral abdominal setae **38, 41** spinnerets, ventral view, arrow in **38** indicating sclerite in front of tracheal spiracle **39** detail of setae on sclerite in front of tracheal spiracle **40, 42** detail of anterior lateral spinnerets **43** epigyne, ventral view **44** palp, ventral view **45** detail of palpal claw and thickened distal prolateral seta **46** detail of palpal claw.

**Figures 47–49.**
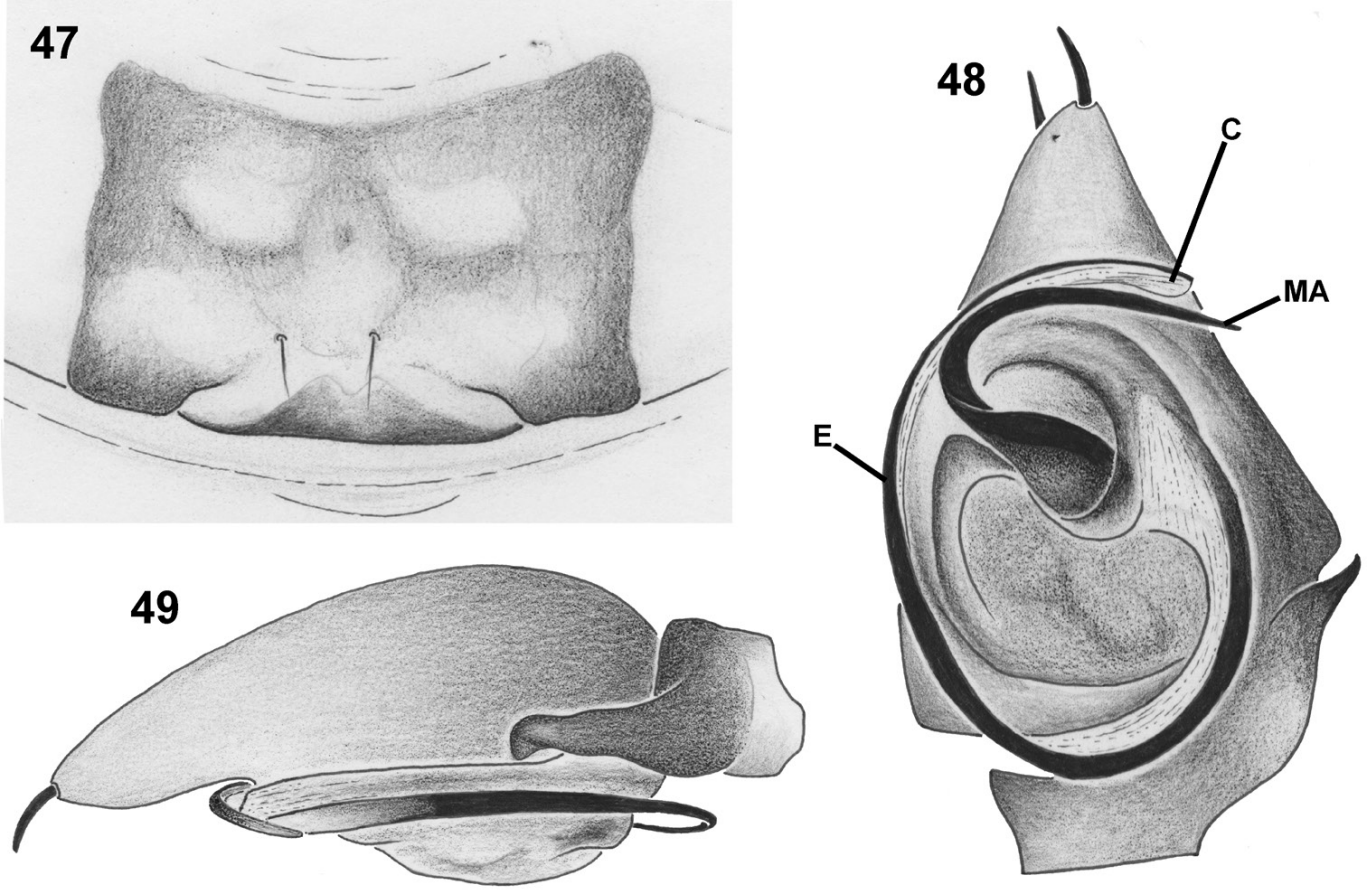
*Mallinusnitidiventris*, female (**47**) and male (**48, 49**) genitalic morphology (NCA 2008/2612) **47** epigyne, ventral view **48** palp, retrolateral view **49** same, ventral view. Abbreviations: C–conductor; E–embolus; MA–median apophysis. Scale bars: 0.1 mm.

**Figures 50–53.**
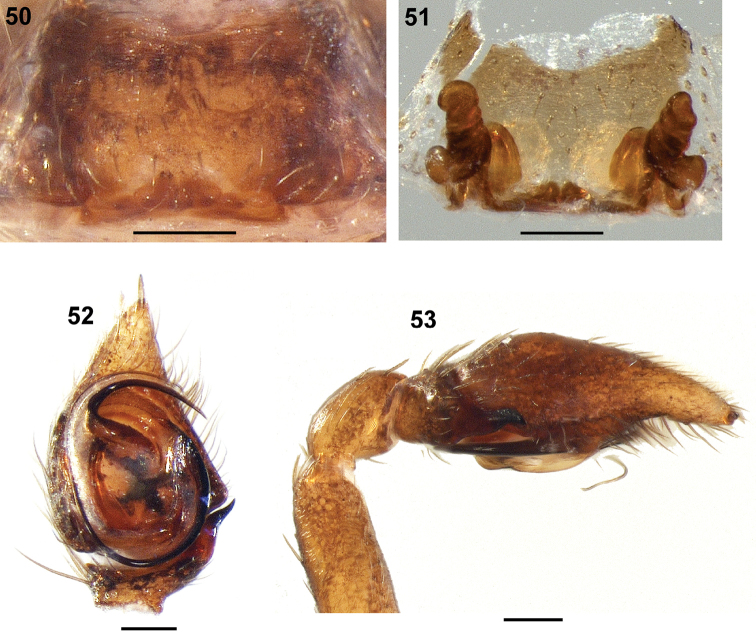
*Mallinusnitidiventris*, female (**50, 51**) and male (**52, 53**) genitalic morphology (NCA 2008/2612) **50** epigyne, ventral view **51** same, digested, dorsal view **52** palp, ventral view **53** same, retrolateral view. Scale bars: 0.1 mm.

Small spiders, 2.13–2.72 mm in length. Carapace longer than wide, with cephalic region similar in length to thoracic region; cephalic region rounded anteriorly, parallel-sided laterally, thoracic region almost circular, broadest at middle of coxa II (Figs 1, 2, 5); cephalic width about 0.77 times thoracic width in males and 0.88 times maximum width in females (cephalic width measured on posterior tangent of PME); carapace in lateral view with strongly convex clypeus, raised behind PER, highest at coxa I, with steep slope in posterior half (Figs 3, 4); surface deeply granulate, sparsely covered in short straight setae with swollen bases, with scattered small pores (Figs 6–10); fovea slit-like, on posterior slope, at two-thirds carapace length (Figs 2, 5, 9). Eye region reasonably broad, AER procurved, PER strongly procurved (Figs 5, 10), with anterior margin of PME behind posterior margin of PLE; all eyes surrounded by black rings; MOQ width equal anteriorly and posteriorly, slightly longer than wide (Fig. 10). Chilum absent. Chelicerae small, narrowed distally, directed posteroventrally; fangs very short and thick (Fig. 11), with posterior groove that anterior margin of endites fits into (Fig. 12); cheliceral promargin with anteromesal cusp provided with one small tooth; setae scarce, each plastron with two long setae; endites almost parallel-sided, rounded distally, converging at midline in front of labium, apically with scale-like extension (Fig. 13), prolateral edge with field of flattened denticles (Fig. 14); labium subtriangular, broader than long (Fig. 11); sternum shield-shaped, as long as broad (Figs 55, 57), surface deeply granulate, covered in scattered erect setae; pleural bars isolated; precoxal triangles small, distinct; intercoxal sclerites absent between coxae I & II, present between coxae II & III and III & IV. Leg formula 4321 (*contra* 4123 in Jocqué 1991), leg IV clearly longer than others; legs covered in mix of short straight, finely barbed setae and incised setae (Fig. 15); femoral organ present on all legs, with single brush-like, densely barbed seta, lying in faint groove riddled with small pits, similar in structure on all four femora (Figs 16–19); patellae without distinct indentation, with lyriform organ retrolaterally at half their length (Figs 20, 21); metatarsi with dense field of short chemosensory setae dorsally (Figs 22, 23), single distal trichobothrium (Figs 24, 25), scattered longer chemosensory setae and barbed setae; metatarsus stopper present, weakly elevated dorsally (Fig. 25); metatarsi without distinct preening comb, metatarsi II–IV with four longer thicker incised setae distally (Figs 26, 27); tarsi ventrally with paired rows of short needle-like setae; tarsal claws paired, with large teeth on margin facing opposing claw (Figs 28, 29), several trichobothria (Fig. 30), chemosensory setae (Fig. 31), subdistal suture (Fig. 29) and weakly elevated oval tarsal organ (Fig. 32); female palpal tarsus conical, with single large claw with three large teeth, turned inward more than 45° (Fig. 33), palpal patella with lyriform organ retrolaterally (Fig. 34). Abdomen globose, higher than long in females, similar in males, with circumferential folds laterally (Fig. 35) and shiny scutum covering dorsum in both sexes (Figs 1–4); petiole short (Fig. 36); abdominal dorsum with sparse covering of short straight setae with fine brachiae, denser ventrally (Fig. 37); epigastric region weakly sclerotised; venter only with inframamillary sclerite present, transversely broad, with row of flattened setae (Figs 38, 39). Spinnerets: ALS of females (Figs 38, 40) and males (Figs 41, 42) long, conical, with central major ampullate gland spigot surrounded by several piriform gland spigots; PMS and PLS of females not studied by SEM, absent in males. Epigyne simple, with median lobe and two adjacent lateral lobes posteriorly incorporating copulatory openings (Figs 43, 47, 50); entrance ducts short, directed slightly laterally, entering lateral spermathecae with three spirals (Fig. 51). Male palp with unmodified femur and patella; tibia with single sharp triangular RTA, curved distally, extending approximately one-quarter the cymbium length (Figs 44, 48, 49, 52, 53); tarsus with triangular cymbium (Fig. 44), with baso-retrolateral process, with broad distal claw and single modified toothed seta prolaterally (Figs 45, 46); embolus long, whip-like, originating retrolaterally, curving around proximal and prolateral margins of tegulum, fine at distal end, tip associated with narrow membranous conductor; MA originating medially on tegulum, C-shaped, with swollen base but sharp end (Figs 48, 52).

##### Relationships.

In a morphological phylogeny of Zodariidae, Jocqué (1991) placed *Mallinus* in the Zodariinae, as sister group to *Ranops* Jocqué, 1991 + *Zodarion*. However, its position would have been clearer had adult specimens been available for study. This lack resulted in a considerable number of missing entries in the character matrix relating to genitalic morphology. Henrard and Jocqué (unpubl.) include a single male *M.nitidiventris* (from Tswalu Kalahari Reserve, MRAC 216253) in their molecular phylogeny of Zodariidae. Their results suggest that *Mallinus* is most closely related to *Palfuria*.

This sister group relationship is also well supported by morphological characters. *Mallinus* shares with *Palfuria* the deeply granulate tegument of the carapace, the strongly raised cephalic region with a steep slope in the posterior half, the scale-like extensions on the endites, the circumferential folds of the abdomen (this character is conspicuous in males and females when the abdomen is not distended), and the subdistal suture on the tarsi. The subdistal suture was also observed in *Akyttara* Jocqué, 1987 and *Heradida* Simon, 1893, to which they are also closely related. In the phylogeny of Henrard and Jocqué (unpubl.), *Ranops* appears to be placed as the sister group of a clade containing *Akyttara*, *Heradida*, *Mallinus* and *Palfuria*, forming a strongly supported monophyletic group. Those zodariines belong to a monophyletic clade characterized by the presence of a unique femoral organ on the legs (Henrard and Jocqué 2017).

#### Mallinus nitidiventris

Taxon classificationAnimaliaAraneaeZodariidae

Simon, 1893

Figs 1–4 , 5–19 , 20–34 , 35–46 , 47–49 , 50–53 , 54–57

Mallinus nitidiventris Simon, 1893: 436, figs 409–410 (juv.); Jocqué 1991: 136, figs 334–335 (juv.).

##### Type material.

Subadult ♂ lectotype and subadult ♀ paralectotype: SOUTH AFRICA: Western Cape: Matjiesfontein, 33°15'S, 20°40'E, MNHN AR 3280 – examined by Jocqué (1991).

##### Other material examined.

SOUTH AFRICA: *Eastern Cape*: Aberdeen district, Farm Bokvlei, 32°25.8'S, 23°21.0'E, 14.XII.2007, leg. D.H. Jacobs (pitfall traps, karoo), 2♂ (NCA 2008/4662); Aberdeen district, Farm De Pannen, 32°41.4'S, 23°25.8'E, 14.XII.2007, leg. D.H. Jacobs (pitfall traps), 1 imm. 4♂ (NCA 2011/604); Aberdeen district, Farm Juriesfontein, 32°31.8'S, 23°25.8'E, 11.XII.2007, leg. D.H. Jacobs (pitfall traps), 1♂ (NCA 2008/4665); Aberdeen district, Farm Nuwejaarsfontein, 32°57.0'S, 24°23.4'E, 14.XII.2007, leg. D.H. Jacobs (pitfall traps, karoo), 2 imm. 7♂ 1♀ (NCA 2008/2612). *Free State*: Luckhoff district, Farm Bankfontein, 30°04.980'S, 24°54.170'E, 22.I.2015, leg. C. Haddad (base of grass tussocks, wetland margin), 1♂ (NCA 2015/1655); Same locality, 30°04.421'S, 24°53.017'E, 6–8.IV.2015, leg. C. Haddad (hand collecting, Nama Karoo veld), 1♂ 1♀ (NCA 2015/1818), 2♂ 1♀ (S.E.M. preparations); Same locality, 30°04.421'S, 24°53.038'E, 26.III.2017, leg. C. Haddad & R. Booysen (hand collecting, Nama Karoo veld), 3 imm. 5♂ (NCA 2017/1447); Same locality, 30°04.974'S, 24°54.297'E, 2–6.IV.2015, leg. University of the Free State students (pitfall traps, Nama Karoo veld), 1♂ (NCA 2015/2398); Same locality, Research camp, 30°04.421'S, 24°53.013'E, 1185 m a.s.l., 24–26.XI.2015, leg. C. Haddad & R. Booysen (night collecting), 1♂ (NCA 2015/2231). *Northern Cape*: Pofadder, 29°22'S, 19°07'E, 15.X.2006, leg. L. Spangenberg (pitfall traps), 1♀ (MRAC 222264); Tswalu Game Reserve, man-made dam, 27°15'S, 22°27'E, 1176 m a.s.l., 6.II.2005, leg. R. Jocqué (savanna shrubland, by hand), 1♂ (MRAC 216253). *Western Cape*: Beaufort West district, Farm Eerste Water, 32°41.4'S, 22°57.6'E, 6.XII.2007, leg. D.H. Jacobs (pitfall traps, karoo), 1 imm. (NCA 2008/2610); Prince Albert, Tierberg, 33°13'S, 22°02'E, 23.IX.1989, leg. R. Dean (old lands), 1♂ (NCA 91/1299), 1♀ (NCA 91/1300).

##### Unconfirmed record.

SOUTH AFRICA: *Northern Cape*: Goepag Nature Reserve, 29°39.906'S, 17°59.838'E, 14–16.VII.2017, leg. R. Booysen, Z. Mbo & R. Christiaan (pitfall traps, Nama Karoo veld), 1 imm. (NCA 2017/1189).

**Female (Bankfontein, NCA 2015/1818). Measurements**: CL 1.32, CW 0.89, AL 1.39, AW 1.36, AH 1.65, TL 2.60 (2.13–2.70), SL 0.62, SW 0.63, AME–AME 0.06, AME–ALE 0.03, ALE–ALE 0.25, PME–PME 0.09, PME–PLE 0.09, PLE–PLE 0.31, MOQAW 0.21, MOQPW 0.22, MOQL 0.25.

Length of leg segments: I 0.70 + 0.29 + 0.54 + 0.67 + 0.41 = 2.61; II 0.71 + 0.30 + 0.53 + 0.73 + 0.45 = 2.72; III 0.71 + 0.32 + 0.53 + 0.78 + 0.45 = 2.79; IV 0.92 + 0.32 + 0.68 + 1.00 + 0.48 = 3.40.

Colour: carapace orange-brown, with faint black mottling and striae (Figs 1, 2); chelicerae orange; endites yellow, slightly darker retrolaterally at midpoint; labium orange, cream distally; sternum orange, cream along anterior margin; leg femora orange-brown; patellae yellow proximally, orange brown distally; tibiae and metatarsi light brown proximally, yellow-brown distally; tarsi yellow; palps yellow-brown; abdomen black dorsally, grey ventrally along midline, with large white patches laterally, fused narrowly in ring around anterior of abdomen; spinnerets creamy-yellow. Eyes: AME diameter 1.1 times ALE diameter; AME separated by distance equal to 0.76 times their diameter; AME separated from ALE by 0.4 times AME diameter; clypeus height 4.2 times AME diameter at AME, 4.16 times ALE diameter at ALE; PME and PLE equal in diameter; PME separated by distance equal to 1.15 times their diameter; PME separated from PLE by distance slightly less than 1.21 times PME diameter; CW:PERW = 2.06:1. Legs spineless, covered in short erect setae and incised setae. Abdomen slightly longer than carapace, higher than long or broad, with shiny scutum covering most of dorsum (Figs 1, 2); dorsum sparsely covered in short straight setae, denser on posterior slope and venter. Epigyne as in genus description (Figs 43, 47, 50, 51). Other characters as in genus description.

**Male (Bankfontein, NCA 2015/1818). Measurements**: CL 1.33, CW 0.88, AL 0.98, AW 0.94, AH 0.98, TL 2.34 (2.23–2.72), SL 0.60, SW 0.59, AME–AME 0.06, AME–ALE 0.03, ALE–ALE 0.25, PME–PME 0.08, PME–PLE 0.08, PLE–PLE 0.29, MOQAW 0.21, MOQPW 0.19, MOQL 0.22.

Length of leg segments: I 0.79 + 0.29 + 0.65 + 0.81 + 0.46 = 3.00; II 0.83 + 0.30 + 0.63 + 0.84 + 0.46 = 3.06; III 0.81 + 0.33 + 0.57 + 0.90 + 0.47 = 3.08; IV 1.03 + 0.33 + 0.75 + 1.15 + 0.53 = 3.79.

Morphology and colouration similar to female (Figs 3, 4), except for the following: AME diameter equals 1.16 times ALE diameter; AME separated by distance 0.67 times their diameter; AME separated from ALE by distance 0.38 times AME diameter; clypeus height 3.85 times AME diameter at AME, 4.0 times ALE diameter at ALE; PME diameter equals 0.89 times PLE diameter; PME separated by distance 1.25 times their diameter; PME separated from PLE by distance 1.25 times PME diameter; CW:PERW = 2.17:1. Abdomen relatively smaller than female (Figs 3, 4), shorter than carapace, as high as long, slightly longer than broad, with conspicuous circumferential folds. Palp as in genus description (Figs 44, 48, 49, 52, 53).

##### Variation.

**Figures 54–57.**
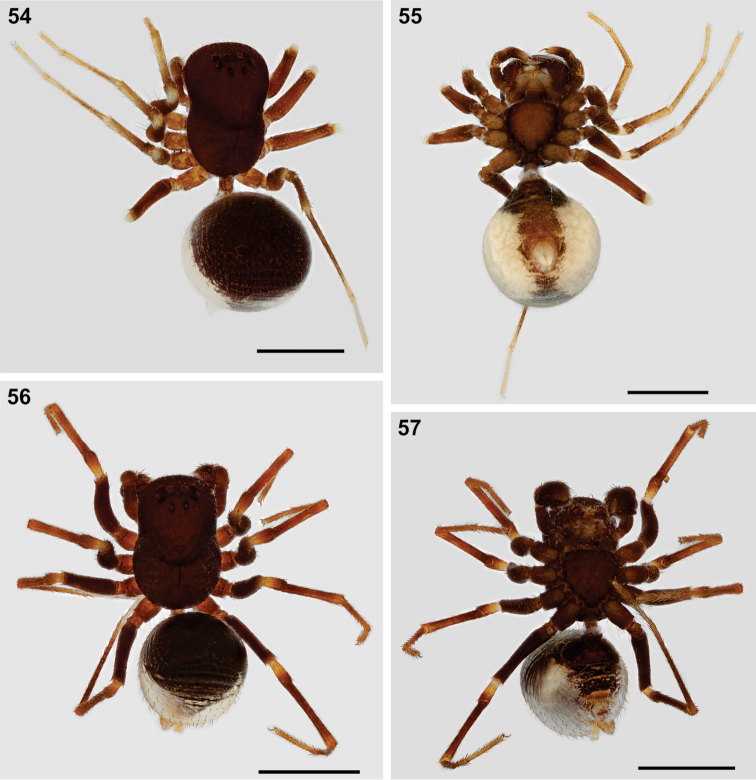
Somatic morphology of *Mallinusnitidiventris* female (**54, 55**) and male (**56, 57**) from Beaufort West, Western Cape (NCA 91/1300 and NCA 2008/2612, respectively) **54, 56** habitus, dorsal view **55, 57** same, ventral view. Scale bars: 1.0 mm.

Populations from the south-western parts of the species’ range (including the type locality) have a clearly darker carapace and legs, which are wine-red in colour (Figs 54–57). It is plausible that populations in this part of its range may associate with a darker species of model ant, affecting their colouration.

**Figures 58–64.**
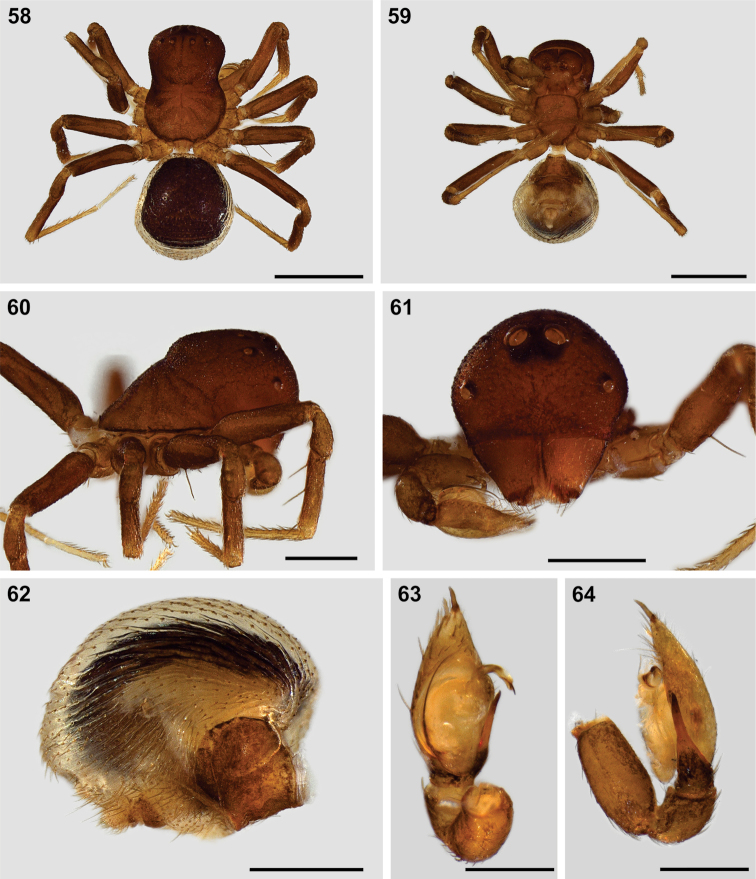
Somatic morphology of “*Mallinus*” sp. male from north-western Namibia (SMN 40843) **58** habitus, dorsal view **59** same, ventral view **60** carapace, lateral view **61** same, anterior view **62** abdomen, lateral view **63** palp, ventral view **64** same, retrolateral view. Scale bars: 1.0 mm (**58, 59**); 0.5 mm (**60–62**); 0.25 mm (**63, 64**).

**Figures 65–66.**
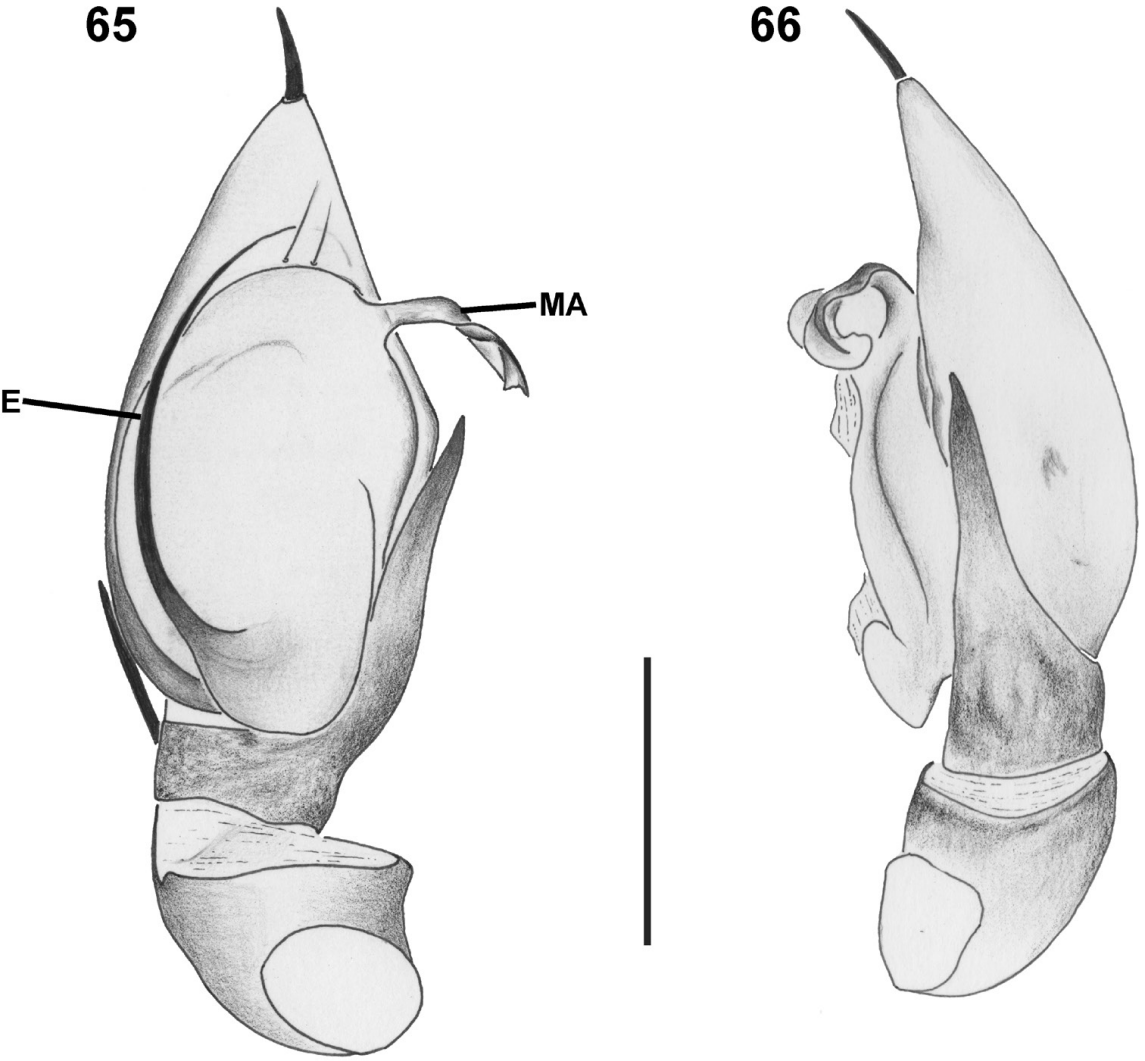
“*Mallinus*” sp., male genitalic morphology (SMN 40843) **65** palp, retrolateral view **66** same, ventral view. Abbreviations: E–embolus; MA–median apophysis. Scale bars: 0.25 mm.

##### Distribution.

**Figure 67.**
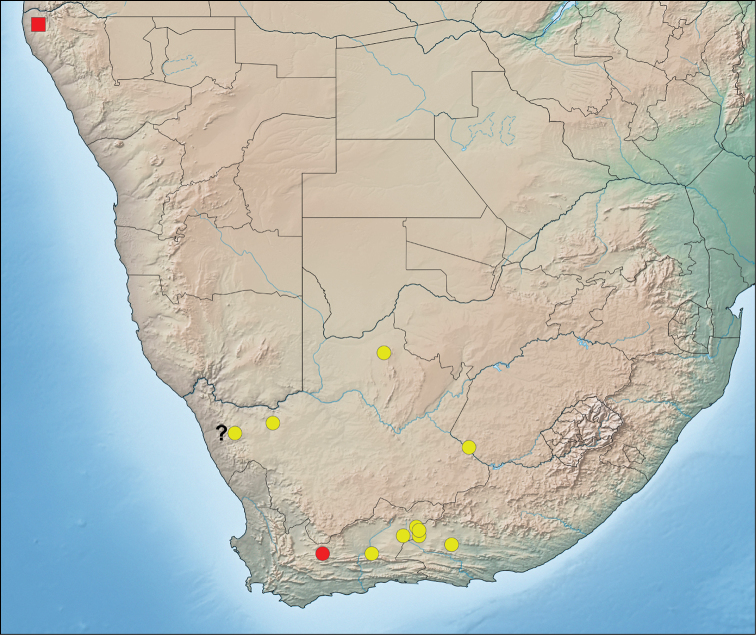
Distribution of *Mallinusnitidiventris* (type locality, red circle; other localities, yellow circles) and “*Mallinus*” sp. (red square) in southern Africa. ? indicates immature specimen requiring confirmation.

Widespread in the western half of South Africa, known from the Eastern Cape, Western Cape, Northern Cape and Free State Provinces (Fig. 67).

##### Habitat and biology.

*Mallinusnitidiventris* is widespread in the semi-arid and arid western half of South Africa, with records in the Nama Karoo and Succulent Karoo biomes, extending into the arid savannas of the southern Kalahari Desert. Specimens collected at Bankfontein in the western Free State Province were all found in Nama Karoo scrubland, either along a hillside or open plains. The substrate at both sites comprised fine Ecca Shale alluvium, siltstone and sandstone gravels that form part of the Ecca Group of the Karoo Supergroup (A. Odendaal and J. Fourie, pers. comm.). Some of the Bankfontein specimens (NCA 2015/1818) were collected during mid-morning (10:00–12:00) foraging in open ground in the vicinity of various ants, including *Anoplolepiscustodiens* F. Smith, 1858, *Camponotus* spp., *Messor* sp. and *Monomorium* sp. Of these, *Mallinusnitidiventris* most closely resembled *Messor* sp. in terms of colouration, although workers of this ant were almost double the body length of the spiders. Only one of these five spiders sampled at this specific site was feeding, a female consuming a *Monomorium* worker ant that measured approximately 2 mm in length, suggesting that this species is myrmecophagous, as are most Zodariinae.

#### “ Mallinus

Taxon classificationAnimaliaAraneaeZodariidae

” sp.

Figs 58–64

##### Material examined.

NAMIBIA: remote place in north-west Namibia about 50 km from coast, 17°37'S, 12°12'E, 13–16.X.1988, leg. E. Griffin (pitfall traps), 1♂ (SMN 40843).

##### Remarks.

We have included the description of a second species in this paper based on a single poorly preserved male from Namibia without formally naming it, as it shows several clear differences to *M.nitidiventris* that make its generic placement dubious: 1) the lack of a palpal conductor on the palp, and 2) the AME that are much larger than the others, and the ALE that are very wide apart and situated in the far lateral corners of the clypeus. This species most likely represents a new genus, and we hope that its description and illustration here will encourage researchers to find fresh material of both sexes and describe and diagnose this taxon thoroughly.

**Male (Namibia, SMN 40843). Measurements**: CL 1.35, CW 0.92, AL 1.25, AW 1.18, AH 1.12, TL 2.70, SL 0.71, SW 0.64, AME–AME 0.05, AME–ALE 0.25, ALE–ALE 0.69, PME–PME 0.18, PME–PLE 0.13, PLE–PLE 0.53, MOQAW 0.33, MOQPW 0.30, MOQL 0.28.

Length of leg segments: I 0.82 + 0.33 + 0.51 + 0.66 + 0.44 = 2.76; II 0.77 + 0.34 + 0.56 + 0.77 + 0.49 = 2.93; III 0.79 + 0.33 + 0.51 + 0.80 + 0.48 = 2.91; IV 1.00 + 0.36 + 0.74 + 1.15 + 0.54 = 3.79.

Colour: carapace medium brown (Figs 58, 60), with dark area around AME, darker stripe between PME (Fig. 61) and dark radiating striae; chelicerae medium brown; endites and labium pale brown; sternum pale brown, with thin darker margin (Fig. 59); leg femora, patellae and tibiae uniform pale brown; metatarsi and tarsi yellow; abdomen dark sepia dorsally (Fig. 58), pale grey laterally and ventrally, area in front of epigastric furrow yellow; spinnerets creamy-yellow. Carapace: cephalic region very broad, almost as broad as thoracic region (Fig. 58); texture finely granulate, without setae. Eyes: AME largest, diameter 1.9 times ALE diameter; AME separated by distance equal to 0.3 times their diameter; AME separated from ALE by 1.6 times AME diameter; clypeus height 1.9 times AME diameter at AME, 2.9 times ALE diameter at ALE; ALE very far apart, situated in far lateral corners of clypeus (Fig. 61); PME and PLE subequal in diameter; PME separated by distance equal to 3.0 times their diameter; PME separated from PLE by distance slightly less than 2.2 times PME diameter; CW:PERW = 2.06:1. Sternum shield-shaped (Fig. 59), shallowly rebordered along lateral margins, with few scattered short setae. Legs with few spines (spination of specimen probably incomplete: FII v1 III v1; TI v1-1 II v1), with some dispersed short erect setae and incised setae. Abdomen slightly shorter than carapace, almost as high as long or broad, with shiny scutum covering most of dorsum and clear circumferential folds (Fig. 62); dorsum sparsely covered in short straight setae, denser on posterior slope and venter; venter sclerotized in front of epigastric fold, with wide, transverse inframamillary sclerite. Palp with long, straight, strongly tapered, sharp RTA; embolus simple, slightly curved; MA membranous, looping; conductor absent (Figs 63, 64). Other characters as in genus description of *Mallinus*.

**Female unknown.**

##### Distribution.

Only known from a single locality in north-western Namibia (Fig. 67).

## Discussion

Most of the 85 genera in the Zodariidae are known from more than one species or are speciose (e.g. *Mallinella* Strand, 1916 with > 200 species), although 10 genera can be considered monotypic following this revision (World Spider Catalog 2018). *Mallinus*, which is now well defined, appears to be one of the uncommon cases of monotypy. *Mallinusnitidiventris* appears to have a large distribution (Fig. 67), which is also a rare phenomenon in the subfamily Zodariinae. Although it is presently known only from the more arid western half of South Africa, it may possibly also occur in nearby southern Botswana and Namibia. At least for the latter, we could not confirm its occurrence in the country, as the collection of the State Museum in Windhoek, Namibia has no invertebrate curator to currently process specimen loans.

The second species, *Mallinusdefectus* Strand, 1906 from Tunisia, was only tentatively attributed to the genus, as Strand (1906) put a question mark behind the genus name: *Mallinus* (?) *defectus*. Considering the predominant distribution patterns of the genera in the Zodariidae, it is most unlikely that the genus occurs both in South and North Africa. It would be the only example of such a distribution. The description of the species by Strand (1906) does not provide a single clue as to the real identity of the genus to which the species belongs. Unfortunately, the type specimens could not be traced, and the species should therefore be considered a ‘*species inquirenda*’.

The distribution of this monotypic genus thus remains exceptionally large, and it is not clear why it has remained like this.

## Supplementary Material

XML Treatment for Mallinus

XML Treatment for Mallinus nitidiventris

XML Treatment for Mallinus

## Supplementary materials

Supplementary material 1 Table S1. Specimen data of *Mallinusnitidiventris* examined in this study Data type: species data Explanation note: This spreadsheet contains all of the available locality and collecting data of *Mallinusnitidiventris* records included in this study. This is an open access article distributed under the terms of the Creative Commons Attribution License (CC BY 4.0), which permits unrestricted use, distribution, and reproduction in any medium, provided the original author and source are credited.Charles R. Haddad, Arnaud Henrard, Rudy Jocqué
